# Type I IFN–Driven Immune Cell Dysregulation in Rat Autoimmune Diabetes

**DOI:** 10.4049/immunohorizons.2100088

**Published:** 2021-10-26

**Authors:** Natasha Qaisar, Adediwura Arowosegbe, Alan G. Derr, Alper Kucukural, Basanthi Satish, Riccardo Racicot, Zhiru Guo, Melanie I. Trombly, Jennifer P. Wang

**Affiliations:** * Department of Medicine, University of Massachusetts Medical School, Worcester, MA; † Diabetes Center of Excellence, University of Massachusetts Medical School, Worcester, MA; ‡ Department of Bioinformatics and Integrative Biology, University of Massachusetts Medical School, Worcester, MA

## Abstract

Type 1 diabetes is a chronic autoimmune disease, characterized by the immune-mediated destruction of insulin-producing β cells of pancreatic islets. Essential components of the innate immune antiviral response, including type I IFN and IFN receptor (IFNAR)–mediated signaling pathways, likely contribute to human type 1 diabetes susceptibility. We previously showed that LEW.1WR1 *Ifnar1*^−/−^ rats have a significant reduction in diabetes frequency following Kilham rat virus (KRV) infection. To delineate the impact of IFNAR loss on immune cell populations in KRV-induced diabetes, we performed flow cytometric analysis in spleens from LEW.1WR1 wild-type (WT) and *Ifnar1*^−/−^ rats after viral infection but before the onset of insulitis and diabetes. We found a relative decrease in CD8^+^ T cells and NK cells in KRV-infected LEW.1WR1 *Ifnar1*^−/−^ rats compared with KRV-infected WT rats; splenic regulatory T cells were diminished in WT but not *Ifnar1*^−/−^ rats. In contrast, splenic neutrophils were increased in KRV-infected *Ifnar1*^−/−^ rats compared with KRV-infected WT rats. Transcriptional analysis of splenic cells from KRV-infected rats confirmed a reduction in IFN-stimulated genes in *Ifnar1*^−/−^ compared with WT rats and revealed an increase in transcripts related to neutrophil chemotaxis and MHC class II. Single-cell RNA sequencing confirmed that MHC class II transcripts are increased in monocytes and macrophages and that numerous types of splenic cells harbor KRV. Collectively, these findings identify dynamic shifts in innate and adaptive immune cells following IFNAR disruption in a rat model of autoimmune diabetes, providing insights toward the role of type I IFNs in autoimmunity.

## INTRODUCTION

Type 1 diabetes (T1D) is a chronic autoimmune disease, characterized by progressive destruction of pancreatic insulin-producing β cells in genetically at-risk individuals. This leads to persistent hyperglycemia, and exogenous insulin replacement therapy is required to achieve normoglycemia (1). Both genetic and environmental factors likely orchestrate an immune-mediated functional loss of β cell mass, leading to the clinical manifestation of disease and lifelong dependence on insulin therapy (2). Viral infections, in particular enteroviruses, have been proposed as causal determinants or initiating triggers for T1D (3). Importantly, virus-induced innate immune responses, particularly type I IFN (IFN-I; IFN-α/β), have been implicated in the initiation of islet autoimmunity and development of T1D (4–6).

Insulitis, defined as an inflammatory lesion of the islets, predominantly consists of cytotoxic CD8^+^ T cells (2), but other infiltrating immune cells such CD4^+^ T cells, B (CD20^+^) cells, macrophages (CD68^+^), NK cells, and neutrophils have been detected in insulitic islets from cadaveric donors (2, 7–10). In contrast, immunosuppressive cells, such as Foxp3^+^ regulatory T (Treg) cells have been rarely detected in insulitic lesions (11) although widely identified in the NOD mouse model (12). We have used the LEW.1WR1 rat model to characterize the role of virus-induced innate immune responses in triggering β cell autoimmunity and T1D pathogenesis (13). Administration of either the dsRNA mimetic polyinosinic:polycytidylic acid (poly I:C) and/or the parvovirus Kilham rat virus (KRV) to weanling rats results in insulitis and diabetes development (14). Diabetes in these rats shares similarities with human disease, including MHC class II association, lack of sex bias, histopathology hallmarks, and juvenile onset of disease (6). LEW.1WR1 rats have normal levels of CD8^+^ T cells, CD4^+^ T cells, and ART2.1^+^ Treg cells (15). Previous studies on diabetes-resistant BB (BBDR) and LEW.1WR1 rats provide evidence for the contribution of T cells in the development of autoimmune diabetes following KRV infection: mAbs against TCR, CD5, and CD8^+^ T cells protect against diabetes, whereas depletion of RT6.1^+^ Treg cells increases the frequency of KRV-induced diabetes (16).

We previously reported that functional disruption of IFN-I signaling through the IFNAR protects rats from poly I:C– or KRV-induced insulitis and diabetes, suggesting that early innate immune responses are critical for diabetes development (13). We further hypothesized that IFNAR deficiency provides protection against diabetes by altering the levels of innate and adaptive immune cells preceding diabetes development. To this end, we examined frequencies of immune cell populations and transcriptome expression in the spleen during the preinsulitic stage. We applied flow cytometry, bulk RNA sequencing (RNA-seq), single-cell RNA-seq, and RNA in situ hybridization (ISH) to identify major leukocyte subsets and immune pathways altered in the spleen preceding insulitis in LEW.1WR1 wild-type (WT) and *Ifnar1*^−/−^ rats. We focused on the spleen given its rich source of immune cells including lymphocytes, macrophages, and NK cells and our ability to recover abundant numbers of cells for various assays and because adoptive transfer of rat splenocytes has identified organ-specific autoreactive cells (16–18). Furthermore, the spleen is an active site of KRV infection and replication for driving the immune response (19). In a separate study, we are examining islet transcriptional changes and immune cell recruitment to the pancreas, the target site of autoimmune inflammation (manuscript in preparation). In this study, we identify splenic innate and adaptive immune players in a virus-induced autoimmune diabetes model and establish that disruption of IFNAR signaling dynamically alters such immune cell populations.

## MATERIALS AND METHODS

### Animals and virus

LEW.1WR1 rats (*RT1B/Du*) were obtained from Biomere (Worcester, MA). LEW.1WR1 *Ifnar1*^−/−^ rats were generated as previously described (13). Animals were housed in viral Ab-free conditions, confirmed monthly to be serologically free of rat pathogens (20) and maintained in accordance with institutional and national guidelines (Institute of Laboratory Animal Resources, United States, 1996). All animal experiments were performed following the Animal Research: Reporting of In Vivo Experiments guidelines and the National Institutes of Health guide for the care and use of laboratory animals. KRV-UMass strain was prepared and titrated by plaque assay as previously described (21).

### Virus infection and tissue collection

Weanling LEW.1WR1 WT and *Ifnar1*^−/−^ rats 21–25 d of age of either sex received a single i.p. injection of KRV-UMass strain (1 × 10^7^ PFU) on day 0. Spleens were collected from age-matched mock-infected (i.e., injected with culture media) or KRV-infected rats at 5 d postinfection (dpi). Blood was collected from euthanized rats by cardiac puncture and immediately mixed with heparin (100 U/ml blood) to prevent coagulation after isolation at room temperature. Isolated rat spleens were collected in PBS (Corning, Oneonta, NY) on ice prior to further processing.

### Preparation of rat spleen cell suspensions

A single-cell suspension of rat spleens was obtained by manual mincing and passing through a 40-μm sterile nylon mesh (Thermo Fisher Scientific, Pittsburgh, PA) with a 3-ml rubber syringe plunger. Cells were collected by centrifugation at 1500 rpm for 3 min. The supernatant was discarded, resuspended in PBS, and centrifuged. The supernatant was discarded, and the cells were resuspended in RBC lysis buffer (BioLegend, San Diego, CA) according to the manufacturer’s method. After removal of RBCs, splenocytes were washed twice with PBS, centrifuged, and resuspended in an appropriate volume of cell staining buffer (BioLegend), and viable cells were counted using the TC20 automated cell counter (Bio-Rad Laboratories, Hercules, CA) using the trypan blue staining method. Approximately 1 ml of rat whole blood was harvested by cardiac puncture using a 1-ml insulin syringe precoated with heparin and collected in tubes containing 100 U/ml heparin. RBC lysis was performed using 1× RBC lysis buffer (BioLegend) according to the manufacturer’s instructions. The cell pellet obtained in the final step was resuspended in staining buffer and viable cells were counted.

### Flow cytometry analysis

We applied a single nine-color rat flow cytometry based on the method previously described by Barnett-Vanes et al. (22) with some modifications. Table I provides a list of all Abs and reagents used in this study for flow cytometry. A total of 1 × 10^6^ splenocytes were stained with Live/Dead dye (Thermo Fisher Scientific, Waltham, MA) in PBS to differentiate live and dead cells. After 30-min incubation, cells were washed twice in PBS. Cells were blocked with anti-CD32 to inhibit nonspecific binding in rat samples according to the manufacturer’s instructions. Splenocytes were aliquoted in a 96-well, round-bottom plate and then stained with Abs diluted 1:100 in Cell Staining Buffer (BioLegend, Franklin Lakes, NJ), followed by incubation at 4°C for 30 min. For neutrophils, we independently used two sets of markers: CD43/His48 or RP-1 for nine-color or eight-color staining panels. After incubation, cells were washed and fixed in Fixation Buffer (BioLegend) overnight at 4°C. For nuclear staining of FOXP3, splenocytes were first stained for surface markers as described above, followed by fixation, permeabilization, and staining with anti-rat FOXP3 using a Foxp3 Transcription Factor Staining Buffer Set (Thermo Fisher Scientific) as per the manufacturer’s instructions.

Flow cytometry compensation was performed using Ultra-Comp eBeads (Invitrogen) as compensation controls following the manufacturer’s instructions. Fluorescence minus one (FMO) controls were used for determining gating boundaries to identify positive and negative populations as previously described (23). The flow cytometry data were acquired by running samples on a Cytek Aurora spectral flow cytometer using SpectroFlo software (Cytek Biosciences, Fremont, CA), followed by unmixing before analyzing the acquired data using FlowJo software (BD Biosciences, San Jose, CA).

### Spleen total RNA preparation, bulk RNA-seq library preparation, and sequencing

Weanling LEW.1WR1 WT and *Ifnar1*^−/−^ rats (21–25 d of age, either sex) were each infected with a single i.p. dose of KRV at 1 × 10^7^ PFU on day 0. Spleens were harvested from mock-infected (i.e., injected with culture media) WT rats (*n* = 2) and *Ifnar1*^−/−^ rats (*n* = 2) or KRV-infected WT rats (*n* = 4) and *Ifnar1*^−/−^ rats (*n* = 4) at 5 dpi. Spleens were homogenized in 1 ml TRIzol reagent using TissueRuptor (QIAGEN, Germantown, MD), and total RNA was extracted following TRIzol method (Invitrogen, Waltham, MA). RNA concentrations were quantified using a NanoDrop spectrophotometer (Thermo Fisher Scientific). Approximately 10 μg total RNA for each of the 12 rat samples was treated with TurboDNase (Thermo Fisher Scientific), and rRNA depletion was performed using a Ribo-Zero Gold rRNA removal kit (Illumina, San Diego, CA). For RNA-seq, we prepared strand-specific libraries by following the protocol from Zhang and colleagues (24). The quality of the prepared libraries was confirmed using the Advanced Analytical Technologies Fragment Analyzer through the Molecular Biology Core Lab (UMass Medical School). The 12 libraries were pooled and sequenced with paired-end reads (75 bp each) using Illumina NextSeq 500 according to the manufacturer’s specifications. RNA-seq data can be accessed at the National Center for Biotechnology Information (NCBI) Gene Expression Omnibus repository (https://www.ncbi.nlm.nih.gov/geo/query/acc.cgi) using accession number GSE114322.

### RNA-seq data analysis

All raw sequencing reads were processed using an in-house pipeline (DolphinNext) at UMass Medical School (25). The clean read pairs were aligned to the rat reference genome, and rRNA sequences were filtered from the mapped reads. The RNA-seq by Expectation Maximization (RSEM) method was used to determine the expression levels of genes by providing mapped transcript counts in the RNA-seq reads (26). Differentially expressed genes (DEGs) were identified using DESeq2 (27), and significant differences in genes were identified as fold change >2 between uninfected and virus-infected samples and if the adjusted *p* value was <0.05 (28). Biological functional annotation and associated pathways of detected DEGs were identified using Gene Ontology (GO; http://geneontology.org). These functional enrichment analyses were performed using the rat reference genome and gene identifiers, adjusted *p* values, and fold changes of the DEGs.

### Infection of normal rat kidney cells and rats for ISH

Subconfluent monolayers of normal rat kidney (NRK) cells (CRL-6509; American Type Culture Collection, Manassas, VA) were grown in DMEM supplemented with 10% FBS on the Nunc Lab-Tek II CC^2^ Chamber Slide System (Thermo Fisher Scientific) for 24 h. A total of 5 × 10^4^ NRK cells were infected with either KRV or vesicular stomatitis virus (VSV; VR-1238; American Type Culture Collection) at a multiplicity of infection of 1 for 24 h. Cell fixation was performed with 10% neutral buffered formalin, followed by gentle rinsing with 1× PBS at room temperature. Cells were then dehydrated with serial wash steps of ethanol. The fixed, dehydrated slides were stored at −20°C until used in RNAscope ISH assays.

KRV-infected weanling WT and *Ifnar1*^−/−^ rats were euthanized, and fresh spleens were harvested. Immediately following dissection, tissues were fixed in 10% neutral buffered formalin for 16-32 h at room temperature. Paraffin-embedded sections were sectioned at 5 μm in the UMass Medical School Morphology Core laboratory (www.umassmed.edu/morphology/protocols).

### Design and synthesis of a KRV-specific probe for ISH

To design a KRV-specific probe, we first determined the sequence of our KRV strain. Briefly, primers spanning the genomic sequence of KRV were designed using the Primer-BLAST tool (www.ncbi.nlm.nih.gov/tools/primer-blast). We then performed PCR followed by sequencing to confirm the genomic identity of our KRV strain. By sequence alignment to available GenBank KRV sequences, our KRV strain showed ~89% identity to the KRV genome of GenBank (https://www.ncbi.nlm.nih.gov/genbank/) accession number U79033. KRV-specific 20 ZZ anti-sense probes were custom designed and synthesized with C2 Channel by Advanced Cell Diagnostics. The KRV probe targets the VP1 region spanning 2334–3615 bp, including an 82-bp intergenic region between the coding sequences.

### Multiplex fluorescence ISH

Multiplex fluorescence ISH was performed using the RNA-scope Multiplex fluorescence V2 Kit according to the manufacturer’s instructions (Advanced Cell Diagnostics, Newark, CA) for both fixed cells and formalin-fixed, paraffin-embedded (FFPE) tissues. Briefly, fixed cells were first pretreated by rehydrating with ethanol and 1× PBS washes, followed by treatment with RNAscope hydrogen peroxide and protease III. FFPE tissue sections were baked at 60°C for 1 h. After deparaffinization with xylene and hydration with ethanol, tissue sections were treated with RNAscope hydrogen peroxide, then heated in RNAscope Ag retrieval buffer, followed by digestion with RNAscope proteinase. A target RNA probe for NK cells was designed based on *Nkg7* (NCBI reference sequence NM_133540.1). Target RNA probes for rat *Ins1* and *Gcg* were available from Advanced Cell Diagnostics, catalog no. 413411 and 315471, respectively. Probes were hybridized to pretreated sections or fixed cells for 2 h at 40° C, followed by a series of signal amplification with kit-provided Preamplifier and Amplifier. Fluorescence signal was generated using Opal 520, 570, and 690 dyes (Akoya Biosciences, Marlborough, MA). Sections or fixed cells were counterstained with kit-provided DAPI, mounted, and stored at 4°C until image acquisition.

### Image quantification

For image quantification, we used the Nikon NIS-Elements imaging software, selecting criteria for color threshold, size, and shape of cells. Images were acquired using a Nikon Eclipse Ti series microscope at 20× objective and analyzed with NIS-Elements Imaging software, version 4.13.04. The Automated Spot Detection tool in NIS-Elements was used to detect positive cells following automated background subtraction and Otsu thresholding. The spot detection tool was set to detect all spots with a diameter of 10 μm and at a contrast value of 100. The number of spots per field was quantified using the Automated Measurement Results tool function, which was exported to Excel. A total of five fields per animal section was included in the analysis.

### Single-cell RNA-seq of rat spleens

Spleens from uninfected WT, uninfected *Ifnar1*^−/−^, KRV-infected WT, and KRV-infected *Ifnar1*^−/−^ rats (*n* = 2 per group) were pooled at 5 dpi and processed for single-cell RNA-seq. We used Seq-Well according to published methods (29) to capture single cells on a microwell array. Each microwell has only one bead carrying oligonucleotides that have a cell barcode, unique molecular identifiers (UMIs), and a polyT tail. Each array was loaded with 20,000 cells. Any remaining cells were put in TRIzol and stored at −80°C. After cell lysis, mRNA transcripts were captured by the oligonucleotides on the bead. The cDNA libraries were prepared using Illumina Nextera XT Library Prep Kits and sequenced with Illumina NextSeq 500.

### Computational analysis

#### Genome alignment and transcript quantification.

The Seq-Well paired-end FASTQ files contain the 12-base cell barcode and eight-base UMIs in the R1 read and the 50-base transcript mRNA sequence in the R2 read. These paired-end reads were preprocessed using a custom Python script to extract the cell barcode and UMI from each R1 read and append them as a colon-delimited pair to the corresponding R2 read name. Reads with Ns in either the cell barcode or UMI were discarded. The resulting FASTQ files were then processed through our DolphinNext analysis pipeline (25) as single-ended reads, removing reads from any cell barcode with fewer than 500 reads. The mRNA sequences were aligned to the rat genome as a reference using tophat2 (v2.0.12) with default settings. Gene transcripts were quantified using End Sequencing Analysis Toolkit (ESAT; https://github.com/garber-lab/ESAT). ESAT ignores reads that result from PCR duplication during the library preparation process using the UMI. If reads from the same cell barcode map to the same gene and have the same UMI, they are considered PCR duplicates, and only one is counted. The output of ESAT is an array containing the transcript counts for each gene for each cell.

#### Cell type identification.

Cell type identification was a multistep process using custom R scripts (R v3.5.0) based on the Signaling-SingleCell package (https://github.com/garber-lab/Signalling-SingleCell). All functions referenced refer to that package. The data for each sample were first processed to remove any cells with fewer than 1000 transcripts and cells with >33% of transcripts from mitochondrial genes, which indicate that the cell is dead or dying (30, 31). All samples were combined into a single ExpressionSet data object for further analysis (R/Bioconductor Biobase package). Two thousand genes were selected using the variance-stabilizing transformation gene selection method to reduce noise introduced by low variance and low expression genes, followed by *t*-SNE mapping [dim_reduce() with default parameters] and density clustering [cluster_sc() with method = density and num_clust = 12] using the selected genes to produce an initial segmentation of the cells. We then used the cluster and mapping boundaries to classify the cells into major groups using the expression levels of known marker genes to identify the groups.

#### Differential expression analysis.

Finally, for each identified cell type, we selected the cells from each condition (WT or *Ifnar1*^−/−^, KRV, or uninfected) and performed differential expression analysis (edgeR) to identify genes that were most differentially expressed in each of the following conditions: KRV WT versus uninfected WT, KRV *Ifnar1*^−/−^ versus KRV WT, KRV *Ifnar1*^−/−^ versus uninfected *Ifnar1*^−/−^, and uninfected WT versus uninfected *Ifnar1*^−/−^.

#### Identifying cells with viral transcripts.

To identify cells containing viral transcripts, we applied Magic-BLAST (https://ncbi.github.io/magicblast/) to all sequencing reads using the KRV genome (GenBank U79033.1) as a reference to identify all reads mapping to the KRV genome. The output of Magic-BLAST was processed with a custom Python script to quantify viral transcripts only in cells with more than 1000 gene transcripts and fewer than 33% mitochondrial transcripts, selected in an earlier step. The script uses a list of cell barcodes to match the viral transcript to the cell and uses the UMI to remove PCR duplicates and ensure that only unique viral transcripts are counted.

#### Pathway enrichment analysis.

Pathway enrichment analysis was performed on DEGs between disease states and visualized as a network of enriched GO terms. GO Enrichment was performed using gProfiler gOST as an ordered query of significantly upregulated and downregulated genes using the databases GO biologic process.

#### Data availability.

The single-cell RNA-seq raw FASTQ files, gene by cell transcript counts matrix, and metadata file including the viral transcript count for each cell are deposited in the NCBI Gene Expression Omnibus database (https://www.ncbi.nlm.nih.gov/geo/query/acc.cgi) with accession number GSE176082.

### Statistical analysis

All statistical analyses were performed with GraphPad Prism 9 software (San Diego, CA). Dual comparisons were made with unpaired Student *t* test or Mann–Whitney *U* test, and groups of four were analyzed by one-way ANOVA with Tukey post hoc test. Statistical significance was considered for *p* < 0.05.

## RESULTS

### Disruption of IFNAR signaling decreases splenic CD8^+^ T cell and NK cell levels and increases neutrophil levels following KRV infection

**FIGURE 1.**
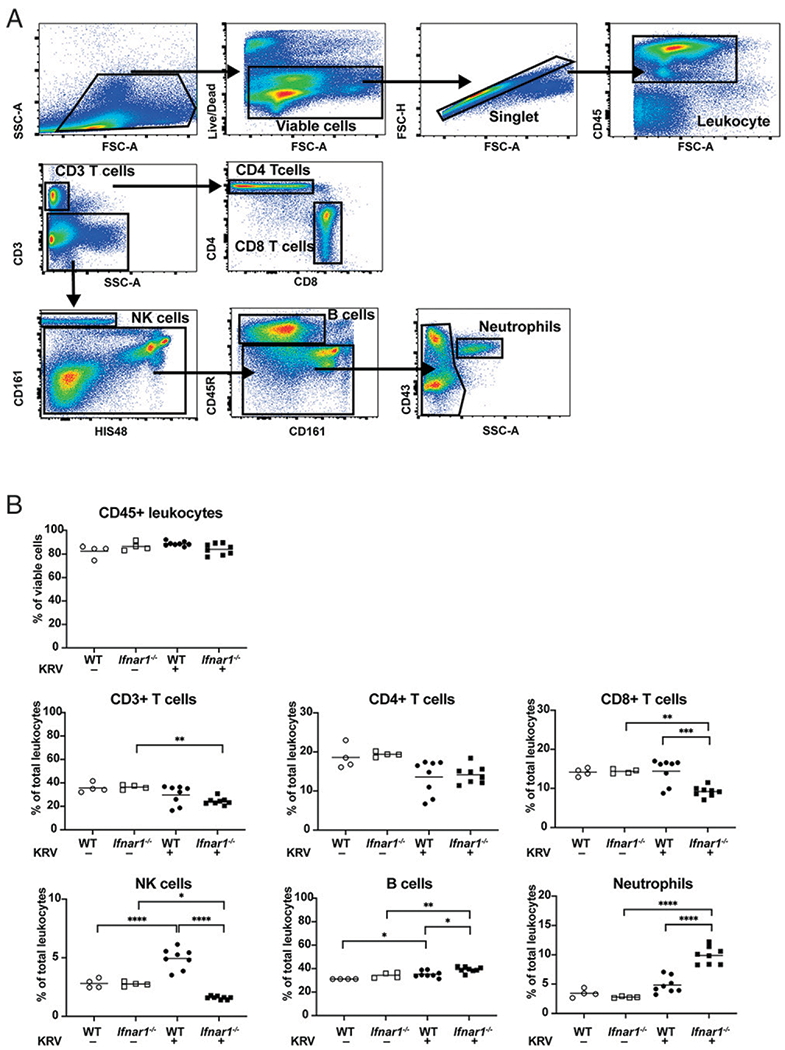
Splenic leukocyte populations shift following KRV infection of WT and *Ifnar1*^−/−^ rats, with decreased CD8^+^ T cells in *Ifnar1*^−/−^ rats, increased NK cells in WT rats, and increased neutrophils in *Ifnar1*^−/−^ rats. (**A**) Representative gating strategies for the flow cytometric analysis of leukocyte subsets in the naive weanling LEW.1WR1 rat spleen. Rat spleens were harvested and processed for flow cytometry with rat-specific mAbs 5 dpi. The viable single cells were gated based on forward light scatter (FSC) and side light scatter. Cells were gated for expression of CD45 and then CD3; CD3^+^ cells were further gated for CD4^+^ or CD8^+^ T cells. Cells lacking CD3 expression were further gated for identifying NK cells (CD161^+^), B cells (CD45R^+^), and neutrophils (CD43^+^) in combination with HIS48 expression and side light scatter. All gating boundaries were established using FMO as described in *Materials and Methods.* (**B**) The percentages of CD45^+^ leukocytes, CD3^+^ T cells, CD4^+^ T cells, CD8^+^ T cells, NK cells (CD161), B cells (CD45R), and neutrophils (CD43) were analyzed using flow cytometry on spleens from age-matched weanling WT and *Ifnar1*^−/−^ rats uninfected or KRV infected at 5 dpi. Each symbol represents a sample from an independently treated animal and the horizontal bar shows the mean value. Data are from *n* = 4 to 8 rats per group from two independent experiments. *p < 0.05, **p < 0.01, ***p < 0.001, ****p < 0.0001, one-way ANOVA with Tukey post hoc tests was used to identify which conditions were significantly different from each other.

We validated the rat-specific nine-color leukocyte panel and gating strategy as described (22) for immunophenotypic analysis of LEW.1WR1 rat spleen by performing flow cytometric analysis with some modifications. Briefly, rat-specific mAbs (Table I) were used to identify major leukocyte subsets in spleens of naive rats by gating first on live, singlet and CD45^+^ cells, which resulted in a live single leukocyte population (Fig. 1A). T lymphocytes were identified by CD3^+^ expression, followed by separation into CD4^+^ and CD8^+^ subsets. NK cells, B cells, and neutrophils were identified by expression of CD161, CD45R, and CD43 with high granularity, respectively.

Our previously published data established that IFNAR deficiency leads to altered cytokine and chemokines during KRV infection (6). Therefore, we tested the possibility that disruption of IFNAR signaling modulates KRV-induced immune responses by altering the frequency of lymphocyte subsets in spleen. For this purpose, we infected weanling WT and *Ifnar1*^−/−^ rats with KRV and harvested spleens and blood at 5 dpi for flow cytometry analysis. Fig. 1B shows the relative proportions of major LEW.1WR1 rat splenic leukocyte subsets identified as the percentage of CD45^+^ cells. At 5 dpi, the percentage of CD3^+^ T lymphocytes in spleen from *Ifnar1*^−/−^ rats was decreased in comparison with uninfected *Ifnar1*^−/−^ rats. No significant difference in the levels of CD4^+^ T cells was observed between the WT and *Ifnar1*^−/−^ rats following KRV treatment. A significant and selective decrease in the percentage of CD8^+^ T cells in spleens from KRV-infected *Ifnar1*^−/−^ rats relative to KRV-infected WT or uninfected *Ifnar1*^−/−^ rats was observed. An increase in the percentage of NK cells was seen in WT rat spleens, but not *Ifnar1*^−/−^ rats, following KRV infection. KRV infection resulted in a slight increase in the percentage of B cells in both WT and *Ifnar1*^−/−^ rats relative to uninfected rats. Our results demonstrate that KRV infection differentially alters the proportions of splenic CD8^+^ T cells, NK cells, and B cells in both WT and *Ifnar1*^−/−^ rats with a specific reduction in the percentage of CD8^+^ T cells and NK cells in KRV-infected *Ifnar1*^−/−^ rats in comparison with KRV-infected WT rats.

Next, we sought to characterize neutrophils in the model. A variety of different surface Ags have been used to identify rat neutrophils (22, 32, 33). We first identified neutrophils in spleens by surface expression of CD43 together with high granularity. This revealed that neutrophils were significantly increased in KRV-infected *Ifnar1*^−/−^ rats relative to uninfected *Ifnar1*^−/−^ or KRV-infected WT rats (Fig. 1B). To independently confirm this finding, we analyzed both blood and spleen for neutrophils from uninfected- or KRV-infected WT or *Ifnar1*^−/−^ rats using anti–RP-1 Ab (32, 33) and the flow cytometry panel and gating strategy shown in Supplemental Fig. 1. KRV induced a significantly increased proportion of neutrophils in both blood and spleen of infected *Ifnar1*^−/−^ rats compared with WT rats at 5 dpi (Supplemental Fig. 1C). Taken together, these data illustrate that the absence of a functional IFNAR signaling system is associated with an increased accumulation of splenic and blood neutrophils following viral infection.

### Splenic Treg cell levels are decreased in WT compared with Ifnar1^−/−^ rats following KRV infection

**FIGURE 2.**
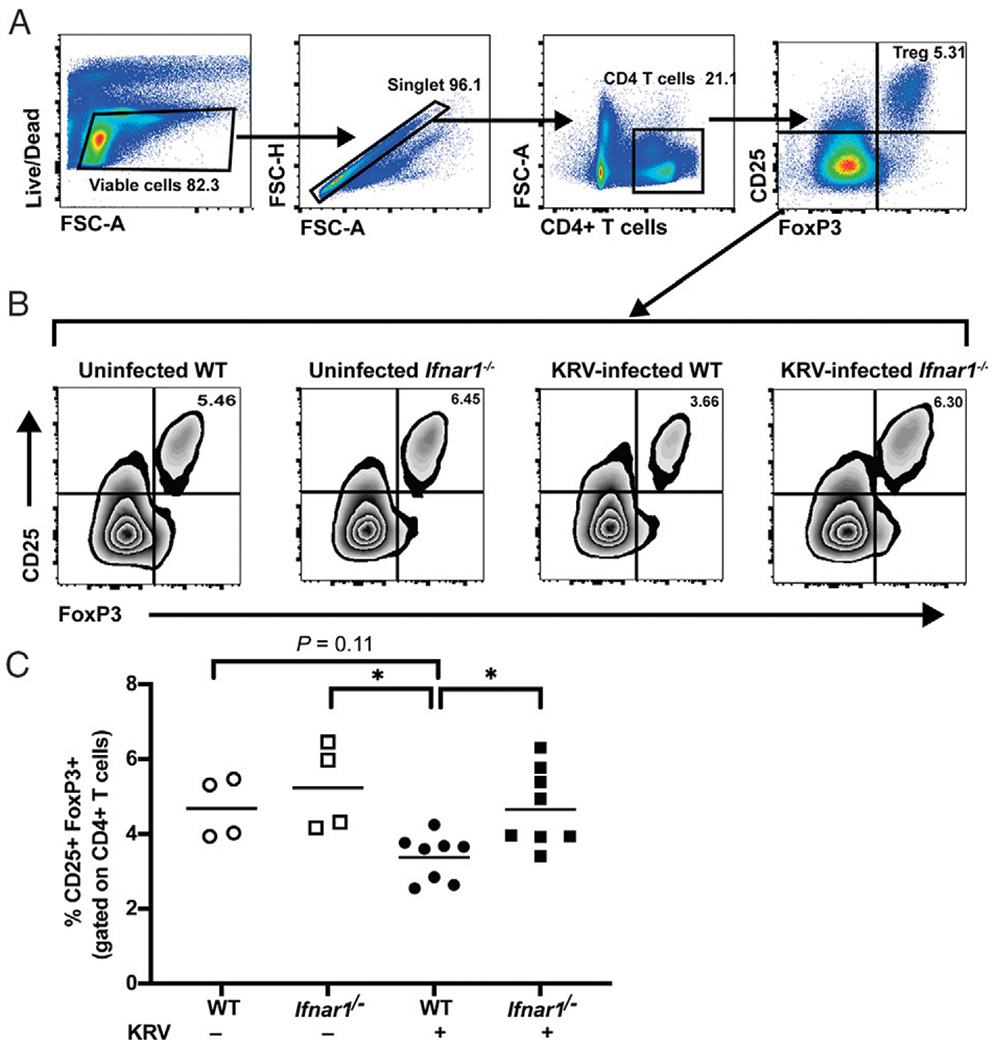
Treg cells are decreased in spleens from KRV-infected WT rats compared with KRV-infected *Ifnar1*^−/−^ rats. (**A**) Representative flow plots of gating strategy for the flow cytometric identification of rat Treg cells in spleens from weanling uninfected versus KRV-infected WT and *Ifnar1*^−/−^ LEW.1WR1 rats. Cells were pregated on forward light scatter (FSC) and side light scatter and further gated on live single CD4^+^ cells. The CD4^+^ cells were then gated for CD25^+^ and FOXP3^+^ coexpression. The percentage of CD25^+^FOXP3^+^ cells is shown on the plot from uninfected WT LEW.1WR1 rat spleens. All gating boundaries were established using FMO as described in *Materials and Methods.* (**B**) Representative flow plots and percentages of CD25^+^ FOXP3^+^ cells from uninfected or KRV-infected LEW.1WR1 WT or *Ifnar1*^−/−^ rats at 5 dpi. (**C**) Quantification of the percentage of Treg cells from rat spleen. The percentage of CD4^+^CD25^+^ FOXP3^+^ Treg cells is significantly reduced in KRV-infected WT rats relative to *Ifnar1*^−/−^ rats at 5 dpi. Each symbol represents a sample from an independently treated animal, and the horizontal bar shows the mean value. Data are from *n* = 4 to 8 rats per group from two independent experiments. *p < 0.05, one-way ANOVA with Tukey post hoc tests was used to identify which conditions were significantly different from each other.

It has been reported that depletion of Treg cells induces diabetes in LEW.1WR1 rats (15). In addition, Treg cells are crucial in controlling pancreatic autoimmunity and maintaining immune tolerance in NOD mice (34). Altered frequency of Treg cells have been reported in children with new onset T1D (35). Thus, we were interested in testing the hypothesis that the mechanism for protective effect against diabetes development mediated through functional disruption of IFNAR signaling involves changes in the frequency of Treg cells. To identify Treg cells in naive weanling rats, we gated live singlet splenocytes for CD4^+^ expression, which were further gated for combined expression of CD25^+^FOXP3^+^. We found ~4–6% of CD4^+^ T cells coexpressing CD25 and FOXP3 in spleen of weanling naive LEW.1WR1 rats, similar to that reported for Sprague Dawley rats, Lewis rats, and Wistar rats (36) (Fig. 2A). We then analyzed Treg populations in WT and *Ifnar1*^−/−^ rats following KRV infection. The proportion of Treg cells trended down in KRV-infected WT rat spleens compared with uninfected WT rat spleens. KRV infection was associated with a significant decrease in the percentage of Treg cells in WT rat spleens relative to *Ifnar1*^−/−^ rat spleens (Fig. 2B). These results demonstrate that IFNAR signaling likely contributes to alteration in the frequency of Treg cells in the spleen during the early stage of diabetes development.

### Transcriptomic analysis of spleens by RNA-seq supports a role for KRV-activated IFN-I signaling pathway in the initiation of autoimmunity

Given the flow cytometry findings, we were compelled to investigate transcriptional changes related to KRV and IFNAR deficiency in our model. To identify candidate genes and pathways involved in regulating immune responses at the early stage of T1D, we employed bulk RNA-seq to compare gene expression profiles in the spleens of WT versus *Ifnar1*^−/−^ rats at 5 dpi. After removing reads aligning to rRNA, the remaining reads were aligned to the rat reference genome by the RSEM method. Based on RSEM estimation, the percentage of aligned reads was (37.7%) for all libraries following removal of multi-mappers. We detected transcripts for at least 49,370 rat genes with a variable read count number per gene for each library based on the RSEM calculation. These data were then further processed for gene expression analysis. We detected differential gene expression levels and their significance levels (*p* values) by using DSEQ by making four comparison groups (1): KRV-infected WT and uninfected WT (2), KRV-infected *Ifnar1*^−/−^ and uninfected *Ifnar1*^−/−^ (3), uninfected WT versus uninfected *Ifnar1*^−/−^, and (4) KRV-infected *Ifnar1*^−/−^ versus KRV-infected WT rats. If the fold change of gene expression was ≥2 and the *p* value was <0.05, the DEGs were identified and further analyzed.

**FIGURE 3.**
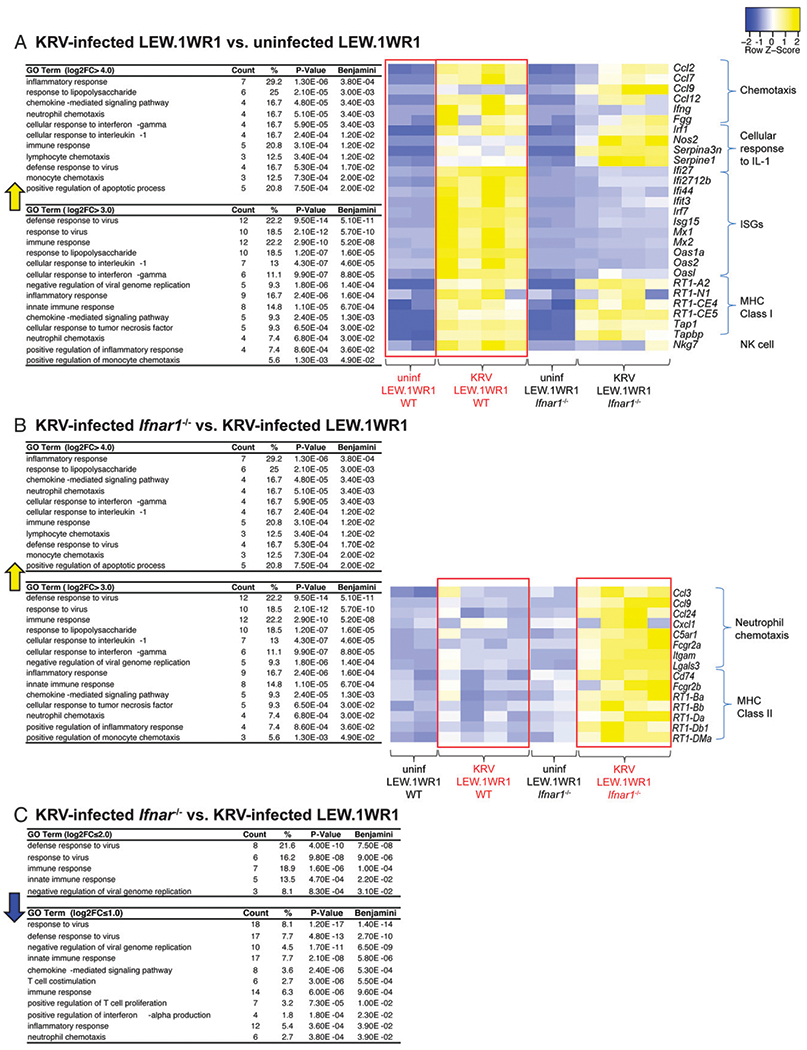
GO analysis of DEGs in rat spleens. (**A**) GO terms for transcripts increased by log_2_ fold change >3.0 in KRV-infected WT compared with uninfected WT rats are shown. The heatmap shows expression trends for select transcripts in individual animals, with each column representing one animal (*n* = 2–4 rats per condition). (**B**) GO terms for transcripts significantly increased by log_2_ fold change >1.0 in KRV-infected *Ifnar1*^−/−^ compared with KRV-infected WT rats. The heatmap shows expression trends for select transcripts in individual animals (one column per animal, *n* = 4 rats per condition). (**C**) GO terms for transcripts decreased by log_2_ fold change <−1.0 in KRV-infected *Ifnar1*^−/−^ compared with KRV-infected WT rats. Benjamini, *p* values after adjusting for a false discovery rate of 5% using the Benjamini–Hochberg method to adjust for multiple hypothesis testing; Count, the number of genes that are in the pathway; %, percentage of the genes in the list that are in the pathway. Yellow arrows indicate increases in the GO terms. Blue arrow indicates decrease in the GO terms. Red boxes highlight compared groups.

We observed different transcript profiles with 474 DEGs for KRV-infected WT and uninfected WT, 1188 DEGs for KRV-infected *Ifnar1*^−/−^ and uninfected *Ifnar1*^−/−^, 128 DEGs for uninfected WT versus uninfected *Ifnar1*^−/−^, and 437 DEGs for KRV-infected *Ifnar1*^−/−^ versus KRV-infected WT rats (Supplemental Table I). GO analysis revealed that spleens from KRV-infected WT rats compared with uninfected WT rats showed strong enrichment for the biological processes for innate and adaptive immune pathways, including those for neutrophil monocytes, neutrophils, and lymphocyte chemotaxis (e.g., *Ccl2, Ccl7, Ccl9, Ccl12,* and *Ifng*), as well as cellular responses to IL-1 (*Fgg, Irf1, Nos2, Serpina3n,* and *Serpinel*) (Fig. 3A). An abundance of IFN-stimulated genes (ISGs) including *Isg15, Mx1, Mx2, Oas1a, Oas2,* and *Irf7* were induced (Fig. 3A, heatmap). In addition, components of Ag processing and presentation by MHC class I represented by *RT1-N1, RT1-CE4, RT1-CE5, RT1-A2, Tap1*, and *Tapbp* were highly induced following KRV infection (Fig. 3A, heatmap). *Nkg7,* a transcript specific for NK cells, was also increased in KRV-infected WT compared with either uninfected WT or KRV-infected *Ifnar1*^−/−^ rat splenocytes, supporting flow cytometry findings shown in Fig. 1A.

GO analysis of transcripts increased in spleens of KRV-infected *Ifnar1*^−/−^ relative to KRV-infected WT rats were striking for enrichment of the cellular response to neutrophil chemotaxis and Ag processing and presentation of Ag via MHC class II. Neutrophil chemotaxis transcripts (*Ccl24*, *CCl3*, *Cxcl1, Fcgr2a, Ccl9, C5ar1, Lgals3,* and *Itgam*) were induced in *Ifnar1*^−/−^ samples compared with WT (Fig. 3B). These bulk RNA-seq results complemented the flow cytometry findings for increased neutrophils in KRV-infected *Ifnar1*^−/−^ rats (see Figs. 1, 3B). The MHC class II transcripts *RT1-Ba, RT1-Bb, RT1-DMa, RT1-Da, RT1-Db1,* and *Fcgr2b* were highly enriched (Fig. 3B). For analysis of those genes induced in KRV-infected WT compared with KRV-infected *Ifnar1*^−/−^ samples, GO analysis showed enrichment in the response to virus and innate immune responses, largely ISGs as expected and displayed in Fig. 3A and 3C.

### Single-cell RNA-seq reveals viral infection of numerous types of immune cells in rat spleens

**FIGURE 4.**
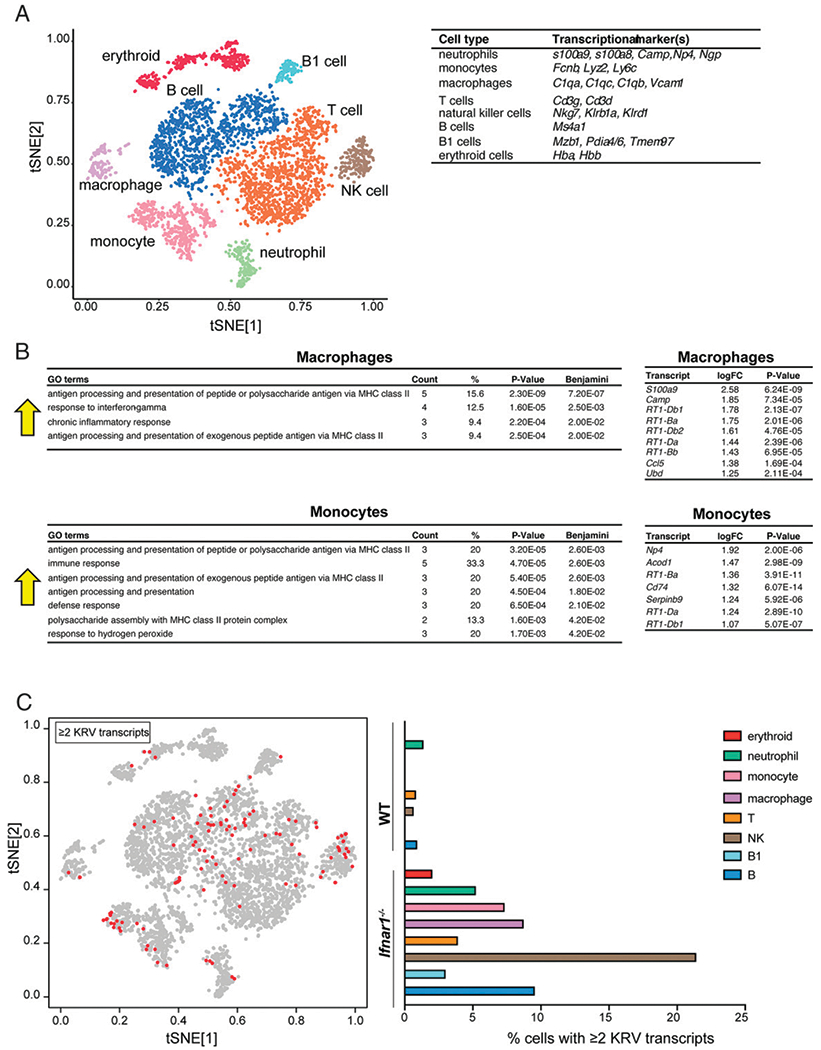
Single-cell RNA-seq analysis reveals increased MHC class II expression in macrophages and monocytes and distribution of KRV transcripts among diverse splenic cell types. (**A**) tSNE plots for rat splenic cells collected at 5 dpi from uninfected WT, uninfected *Ifnar1*^−/−^, KRV-infected WT, and KRV-infected *Ifnar1*^−/−^ rats. Clusters are labeled for eight cell types. Markers for cell identification are indicated. (**B**) GO analysis for macrophage and monocyte transcripts significantly increased by log_2_ fold change >1.0 in KRV-infected *Ifnar1*^−/−^ compared with KRV-infected WT rats. The specific log_2_ fold change for contributing transcripts is displayed. Arrows indicate increases in the GO terms. (**C**) KRV transcripts appear in all types of splenic cells. tSNE plot highlights cells associated with at least two KRV reads (red dots). Data are from *n* = 2 rats per group. Benjamini, *p* values after adjusting for a false discovery rate of 5% using the Benjamini–Hochberg method to adjust for multiple hypothesis testing; Count, the number of genes from the list of genes provided that are in the pathway; logFC, log_2_ fold change; %, percentage of the genes in the pathway.

**FIGURE 5.**
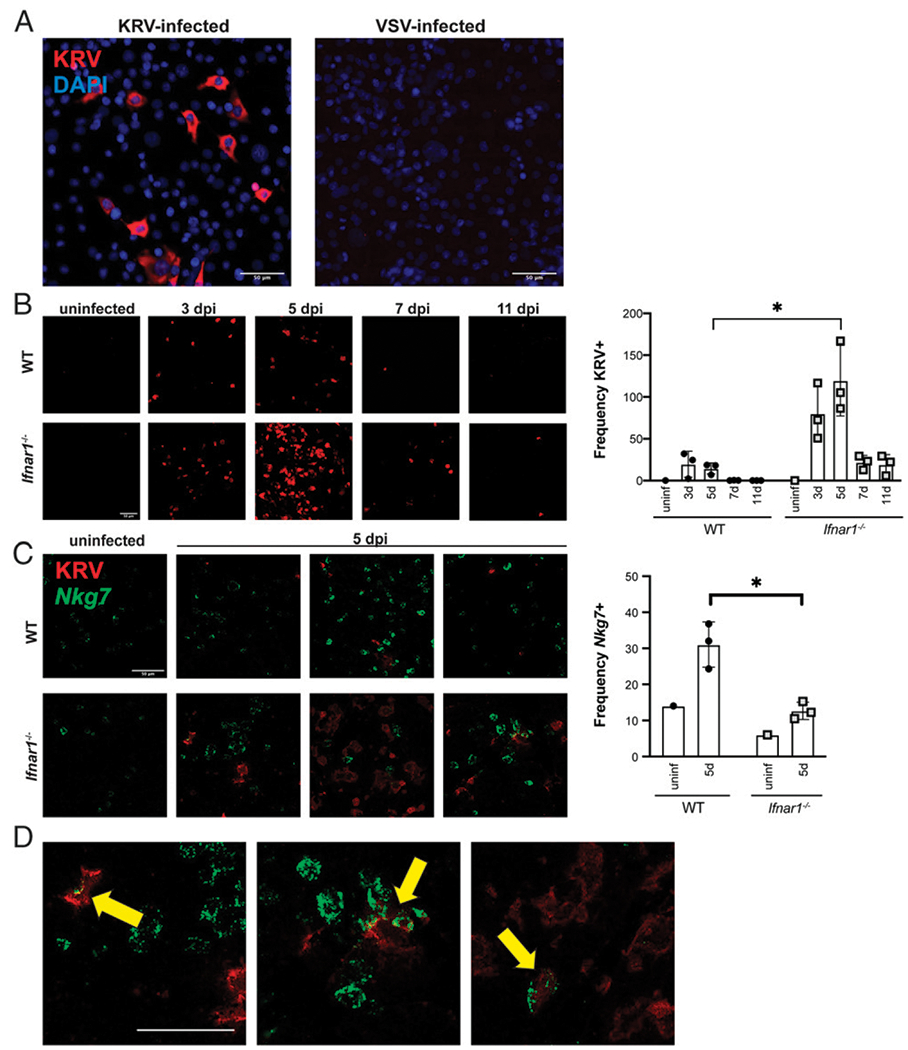
ISH confirms that NK cells are significantly more abundant in KRV-infected WT spleens than in KRV-infected *Ifnar1*^−/−^ spleens and that KRV infects NK cells. (**A**) NRK epithelial cells were infected with KRV at multiplicity of infection (MOI) of 1 for 24 h, then fixed and stained with DAPI and an ISH probe specific for KRV. VSV-infected cells served as a specificity control for the KRV probe. (**B**) ISH for KRV (red) in rat spleens shows that virus is detected at day 3, peaks at day 5, then declines. Images shown are representative of one animal per condition. The plot shows the image quantification average from five fields per animal (uninfected WT, *n* = 1; uninfected *Ifnar1*^−/−^, *n* = 1; KRV-infected WT, *n* = 3; KRV-infected *Ifnar1*^−/−^, *n* = 3 each for days 3, 5, 7, and 11). (**C**) KRV (red) and *Nkg7* (green) ISH staining are shown for day 5 spleens. One representative image from each rat is shown. *Nkg7* is abundant in KRV-infected WT rats. The plot shows the image quantification average from five fields per animal (uninfected WT, *n* = 1; uninfected *Ifnar1*^−/−^, *n* = 1; KRV-infected WT, *n* = 3; KRV-infected *Ifnar1*^−/−^, *n* = 3 for day 5). (**D**) Arrows highlight *KRV*^+^/*Nkg7*^+^ cells. Scale bar, 50 μm. *p < 0.05 with multiple paired *t* tests.

Next, we processed rat spleens (5 dpi) from uninfected and KRV-infected LEW.1WR1 WT and *Ifnar1*^−/−^ rats for single-cell RNA-seq. Libraries were generated using the Seq-Well platform and sequenced on the Illumina NextSeq 500. Samples were filtered to remove cells with <500 transcripts per cell (i.e., too few transcripts for analysis) and >33% mitochondrial transcripts, which are indicative of dead or dying cells. We obtained a total of 4765 cells across all samples (748 for uninfected WT, 688 for uninfected *Ifnar1*^−/−^, 1782 for KRV-infected WT, and 1546 for KRV-infected *Ifnar1*^−/−^). We found a total of 8.4 million transcripts (1.2 million for uninfected WT, 1.2 million for uninfected *Ifnar1*^−/−^, 3.1 million for KRV-infected WT, and 2.9 million for KRV-infected *Ifnar1*^−/−^) with an average of 1766 transcripts per cell and an average of 736 transcripts detected per cell. To detect specific splenic cell populations, we performed *t*-distributed stochastic neighbor embedding (tSNE) mapping and unsupervised density clustering and obtained eight distinct clusters (Figs. 4, 5A). One cluster was identified as erythroid cells based on high expression of hemoglobin genes (*Hba* and *Hbb*). Immune cell lineages were identified based on high expression of classical cell type markers reported in the literature for rats, mice, and humans and included B, B1, T, and NK cells as well as neutrophils, monocytes, and macrophages (see Fig. 4, Supplemental Fig. 2A). Overall differences in the fractions of cell types for each of the four conditions were modest, albeit an increase in the percentage of NK cells in KRV-infected WT rat samples agreed with flow cytometry trends (Supplemental Fig. 2B).

We performed a pathway enrichment analysis by generating gene enrichment maps on DEGs between the found conditions and visualized these pathways as a network of enriched GO terms. Not surprisingly, we identified enriched GO annotations related to the IFN-I response in nearly all cell types for KRV-infected WT compared with uninfected WT samples (Supplemental Table I). For KRV-infected *Ifnar1*^−/−^ samples, anticipated decreases in ISGs were also observed in comparison with KRV-infected WT samples across various cell types (Supplemental Table I). Through differential expression analysis, we identified monocytes and macrophages as the source of highly induced MHC class II transcripts identified in our bulk transcriptome studies for KRV-infected *Ifnar1*^−/−^ compared with KRV-infected WT samples (Fig. 4B).

Previous studies reported that KRV infects rat lymphoid organs including the spleen (19). We examined the various splenic immune cell types associated with at least two viral transcripts per cell by single-cell RNA-seq. KRV transcripts were distributed among all of the immune cell populations, with NK cells from *Ifnar1*^−/−^ rats having the highest percentage of KRV transcripts (Fig. 4C). The overall greater number of KRV-positive cells in *Ifnar1*^−/−^ spleens is likely secondary to the absence of IFN-I signaling, resulting in reduced clearance of the viral transcripts. No KRV sequences were found in spleens from uninfected WT or uninfected *Ifnar1*^−/−^ rats, as expected.

### RNA ISH confirms viral infection of splenic NK cells

To independently confirm that splenic cells support KRV infection, we designed a probe specific for KRV to use for ISH (see Materials and Methods). As shown in Fig. 5A, the probe revealed an abundance of KRV transcripts in KRV-infected NRK epithelial cells. VSV-infected NRK cells were negative for KRV staining, confirming specificity of the probe. Next, spleens from KRV-infected WT and *Ifnar1*^−/−^ rats were harvested at 3, 5, 7, and 11 dpi (*n* = 3 animals per condition); spleens from uninfected WT and *Ifnar1*^−/−^ rats (*n* = 1 animal per condition) were used as negative controls. FFPE spleens were processed for staining with the KRV-specific ISH probe. Fig. 5B shows that KRV levels peak at 5 dpi in the spleen before declining, with averages being higher in *Ifnar1*^−/−^ rats than in WT rats. Images are shown for one representative rat per condition. In Fig. 5C, ISH confirms that KRV infects splenic NK cells (*Nkg7*^+^) and that NK cells are increased in frequency in KRV-infected WT compared with KRV-infected *Ifnar1*^−/−^ rats. Fig. 5D shows representative *Nkg7*^+^/*KRV*^+^ cells that demonstrate that NK cells are infected with KRV.

**FIGURE 6.**
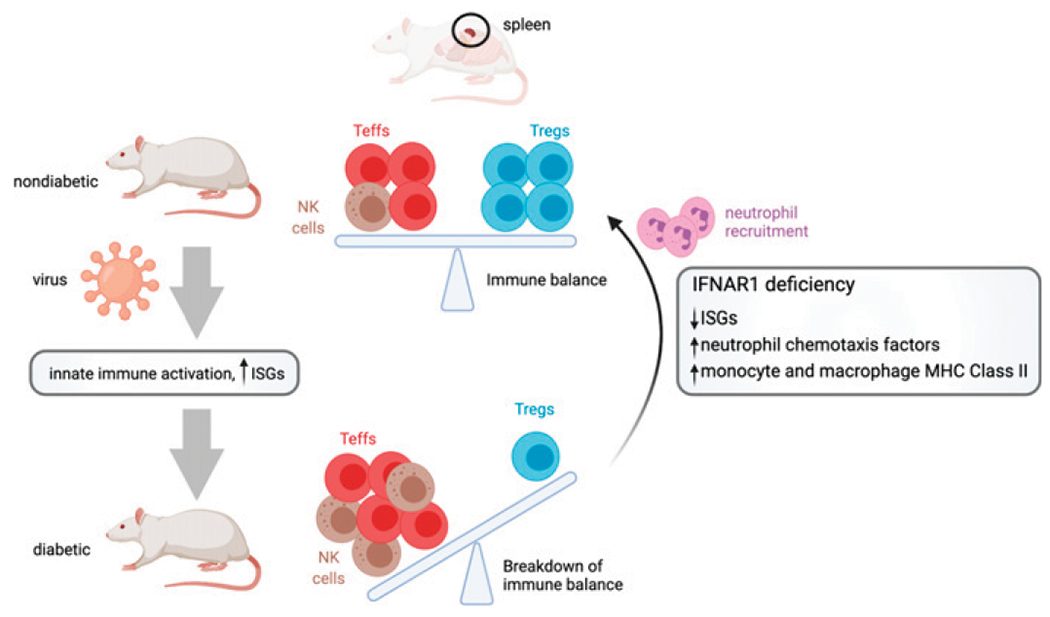
IFNAR1 deficiency restores the balance of innate and adaptive immune cells to protect against autoimmune diabetes. Created with BioRender.com.

Finally, an early study reported that pancreatic islets are not infected with KRV in rat models of autoimmune diabetes, as ISH for KRV sequences in the pancreas revealed that transcripts were only in endothelial and interstitial cells (19). However, a more recent study that used antiviral immunostaining suggested that β cells are infected with KRV (37). To address this discrepancy, we used our KRV-specific ISH probe on FFPE whole pancreas from a WT LEW.1WR1 rat at 5 dpi. By combining this with a rat insulin-specific probe, we concluded that rat β cells are not directly infected with KRV (Supplemental Fig. 3). Our collective study findings are summarized in Fig. 6.

## DISCUSSION

To better define immune cell processes that precede the onset of autoimmune diabetes, we used a model in which viral infection triggers diabetes and in which absence of IFN-I signaling protects weanling rats from disease. The exact mechanism for KRV-induced diabetes in rats is not fully understood, but it may involve virus-induced alterations in the immune-regulatory system such as activation of innate and adaptive immune responses with reduced Treg cell frequency in the lymphoid organs including spleen and pancreatic lymph nodes (6, 21, 38–41). We focused on splenocytes given that adoptive transfer of Con A–activated spleen cells from KRV-infected rats into congenic rats leads to diabetes and insulitis (16) and that KRV infects the spleen (19).

We performed flow cytometry and transcriptional analysis of splenocytes collected from LEW.1WR1 rats prior to the onset of insulitis and autoimmune diabetes under four conditions: WT without virus (no disease), *Ifnar1*^−/−^ without virus (no disease), WT with KRV (diabetes), and *Ifnar1*^−/−^ with KRV (reduced incidence of diabetes). KRV-infected WT rats have increased levels of splenic NK cells and decreased levels of Treg cells in comparison with uninfected WT rats, with overall increases in IFN-I and inflammatory pathways at the transcriptional level. The proportions of NK cells and Treg cells are similar between KRV-infected *Ifnar1*^−/−^ and uninfected WT rats. Furthermore, cytotoxic CD8^+^ T cell levels are decreased in KRV-infected *Ifnar1*^−/−^ rats relative to KRV-infected WT rats. Likewise, Treg cells are found in lowest proportions in WT rats following KRV infection. Transcriptional analysis reveals elevated neutrophil chemotactic factors and the induction of MHC class II in monocytes and macrophages from KRV-infected *Ifnar1*^−/−^ rats. In agreement with the high expression of neutrophil chemotactic factors, neutrophil proportions are increased in splenocytes and in peripheral blood leukocytes of KRV-infected *Ifnar1*^−/−^ rats compared with KRV-infected WT rats. These findings are summarized in Fig. 6 and underscore the contributions of both innate and adaptive immune cells to the balance hypothesis in autoimmune diabetes (42).

NK cells are innate lymphocytes that mediate several antimicrobial and antitumor properties (43). Activated NK cells exert direct cytolytic effects against infected cells by releasing perforin and granzyme and through their interactions with the innate and adaptive immune systems by producing several proinflammatory cytokines including IFN-γ and TNF (44). NK cells mediate an effective immune response by interacting with ligands present on target cells via their inhibitory and activating receptors (45). Consequently, aberrant expression of NK receptor ligands may trigger or exacerbate deleterious effects of NK cells in autoimmunity, infections, and cancer settings (44, 46). Several studies have reported the correlation between altered NK cell number and/or function in T1D patients (47–50). Mice deficient in NK cell activating receptor, NKp46, have reduced development of T1D (51). Rat NK cells express receptor protein 1 (NKR-P1; also called CD161), a homodimeric 30-kDa type II transmembrane C-type lectin, which is also expressed on a subset of T cells, dendritic cells, and activated monocytes (52, 53). Depletion of both cytotoxic T cells and NK cells BB-diabetes prone rats conferred protection from diabetes and raised the possibility that NK cells participated in autoimmune disease (54). However, the exact role of NK cells in human T1D remains ill defined (55). The absence of elevated NK cells in KRV-infected *Ifnar1*^−/−^ rats is consistent with known requirements of IFN-I for activation of NK cells in response to viral infections (56–58).

Macrophages were shown in previous studies to play an important role in KRV-induced diabetes in BBDR rats (59). Increases in MHC class II expression were reported in BBDR rat splenic macrophages within the week following poly I:C and KRV injection (60). Our finding of heightened macrophage MHC class II expression in the context of IFNAR1 deficiency links MHC class II expression with the IFNAR signaling pathway, but the direct cause and consequences of this remain unclear and deserve attention in future studies.

Neutrophils are the most abundant leukocyte subset in the human peripheral blood circulation and are the primary responders against invading microbes at the sites of infection and injury and initiate an acute inflammatory response (61). They are classically known to exert their innate immune functions through phagocytosis through production of reactive oxygen species, degranulation, and formation of neutrophil extracellular traps—a network of structures composed of chromatin bound to neutrophil granular antimicrobial peptides—which are released during a process called NETosis (61–63). Renewed interest in their functional diversity has revealed novel functions of neutrophils such as the production of cytokines and chemokines, as well as interaction with endothelial cells, dendritic cells, macrophages, NK cells, T cells, and B cells to regulate both innate and adaptive immune responses (64). As such, aberrant functions of neutrophils have been recognized to contribute to the development of several autoimmune disorders (62, 64). Diana et al. (65) first demonstrated that in young female NOD mice, physiological β cell death initiates the recruitment and activation of neutrophils, B-1a cells, and plasmacytoid dendritic cells to the pancreas, with the involvement of plasmacytoid dendritic cell–derived IFN-α and neutrophil-derived, cathelicidin-related antimicrobial peptide. However, diabetes can develop in NOD mice in the absence of neutrophils (66). A reduction in circulating neutrophil numbers has been observed preceding T1D disease onset and pancreas-residing neutrophils extrude neutrophil extracellular traps. In our model, proportions of neutrophils between uninfected and KRV-infected WT rats are similar, and the increased proportion of neutrophils in KRV-infected *Ifnar1*^−/−^ rat spleens appears to be a feature of viral infection in the absence of IFNAR1 and is not necessarily associated with the development of autoimmunity. Evaluation of pancreatic tissue is needed to determine if any changes in infiltrating neutrophils are detected.

Our study highlights the importance of the innate arm of the immune system in the prediabetes stage. By understanding such processes, we may better define mechanisms and therapeutic interventions specific to innate immunity to prevent T1D (67). We capture the dynamic interplay between innate and adaptive immunity in the T cell lineage findings, defining relative changes in cytotoxic CD8^+^ lymphocytes, NK cells, and Treg cells in *Ifnar1*^−/−^ rats that are protected from diabetes following viral challenge. We also demonstrate viral infection of specific splenic cells and identify transcriptional signaling pathways associated with chemotaxis and inflammation in the prediabetic stage. Depletion or adoptive transfer of specific cell populations such as NK cells in rat models of diabetes can help establish their essential roles in autoimmune disease. Exploration of other immune cell populations such as type 1 regulatory cells in this model may identify their contributions to autoimmune diabetes. Correlation between immune cell alterations in the spleen to that in the pancreas as well as other nonendocrine tissues (e.g., lymph nodes and thymus), will help reveal pathologic roles for such cells in autoimmune diabetes.

## Supplementary Material

Supplemental File 1

Supplemental Material

## Figures and Tables

**TABLE I.** Rat-specific mAbs used in flow cytometry

Ab/Reagent	Fluorochrome	Clone	Dilution	Vendor
Nine-color staining panel				
Live/Dead	eFluor 780	N/A	1:1000	eBioscience
CD32	N/A	D34-485	1:200	BD Biosciences
CD45	Pacific Blue	OX-1	1:200	BioLegend
CD3	Allophycocyanin	1F4	1:100	BioLegend
CD4	PerCP-eFluor710	OX-35	1:100	eBioscience
CD8	PE-Cy5	OX-8	1:100	Invitrogen
CD161	PE	3.2.3	1:200	BioLegend
CD45R	PE-Cy7	HIS24	1:100	eBioscience
CD43	PerCP-Cy5.5	W3/13	1:100	BioLegend
His48	FITC	HIS48	1:00	eBioscience
Five-color staining panel				
Live/Dead	eFluor 780	N/A	1:1000	eBioscience
CD32	N/A	D34-485	1:200	BD Biosciences
CD45	Pacific Blue	OX-1	1:200	BioLegend
CD3	Allophycocyanin	1F4	1:100	BioLegend
CD45R	PE-Cy7	HIS24	1:100	eBioscience
RP-1	PE	RP-1	1:100	BD Biosciences
Treg staining panel				
CD4	FITC	OX-35	1:100	BioLegend
CD25	PE	OX-39	1:100	BioLegend
FOXP3	eFluor 450	FJK-16s	1:100	eBioscience

## Abbreviations used in this article:

BBDR: diabetes-resistant BB
DEG: differentially expressed gene
dpi: day postinfection
ESAT: End Sequencing Analysis Toolkit
FFPE: formalin-fixed, paraffin-embedded
FMO: fluorescence minus one
GO: Gene Ontology
IFN-I: type I IFN
ISG: IFN-stimulated gene
ISH: in situ hybridization
KRV: Kilham rat virus
NCBI: National Centerfor Biotechnology Information
NRK: normal rat kidney
poly I:C: polyinosinic:polycytidylic acid
RNA-seq: RNA sequencing
RSEM: RNA-seq by Expectation Maximization
T1D: type 1 diabetes
Treg: regulatory T
tSNE: *t*-distributed stochastic neighbor embedding
UMI: unique molecular identifier
VSV: vesicular stomatitis virus
WT: wild-type
