# Supplementary material for: Type I IFN–Driven Immune Cell Dysregulation in Rat Autoimmune Diabetes
Source: Immunohorizons. Author manuscript; Available in PMC 2022 May 16. (PMC9109816; doi:10.4049/immunohorizons.2100088)
Supplement: Supplemental Material [file NIHMS1802390-supplement-Supplemental_Material.docx]

**SUPPLEMENTAL MATERIALS FOR:**

**Type I interferon-driven immune cell dysregulation in rat autoimmune diabetes**

Natasha Qaisar*†, Adediwura Arowosegbe*†, Alan G. Derr †‡, Alper Kucukural‡, Basanthi Satish*†, Riccardo Racicot*, Zhiru Guo*, Melanie I. Trombly*, and Jennifer P. Wang*†

**
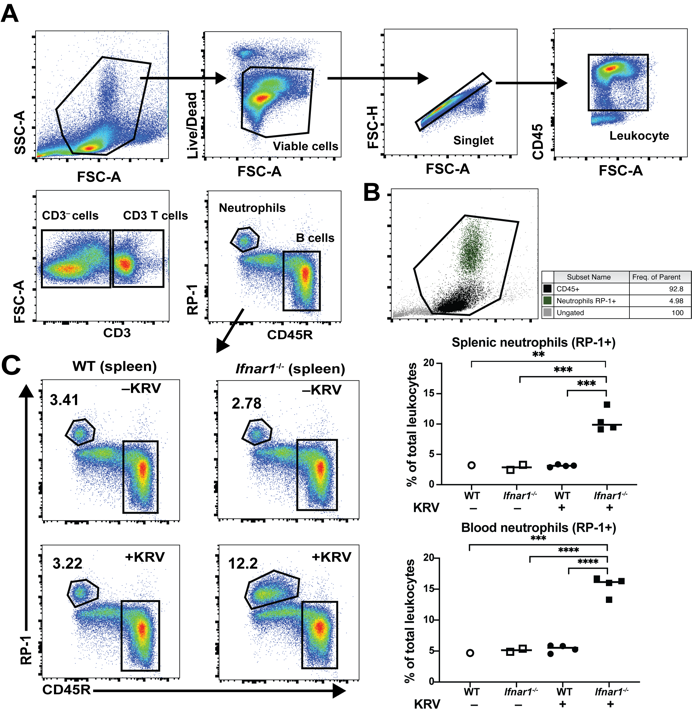
**

**Supplemental Figure 1. Neutrophils are increased in KRV-infected LEW.1WR1 *Ifnar1^-/-^* rat spleens and peripheral blood by RP-1 staining. (A)** Representative gating strategies for flow cytometric analysis of neutrophils in weanling LEW.1WR1 rat spleens. Rat spleens and blood collected at 5 dpi with KRV were analyzed by flow cytometry analysis with rat-specific monoclonal antibodies. Rat neutrophils were stained for monoclonal antibody targeting surface antigen RP-1. The viable single cells were gated based on FSC and SSC and further discriminated based on CD3 expression. CD3- cells were then gated for RP-1 expression. The CD45R marker was additionally used to help in separating RP-1+ cells. All gating boundaries were established using FMO as described in the Methods section. A similar flow cytometric analysis was performed for the rat peripheral blood (not shown). (**B)** Representative flow cytometric analysis of rat spleen leukocytes (CD45+) by FSC and SSC characteristics. Neutrophils from spleen were back-gated to show high SSC profile based on specificity of RP-1 expression. **(C)** Sample flow plots examining RP-1 and CD45R expression on rat splenocytes from LEW.1WR1 WT and *Ifnar1^-/-^* rats 5 dpi (left panel). Quantification of the percentage of RP-1+ neutrophils from rat spleen and peripheral blood (right panel). RP-1+ neutrophils are significantly increased in KRV-infected *Ifnar1^-/-^* rats 5 dpi. Each symbol represents a sample from an independently infected animal, and the horizontal bar shows the mean value. One-way analysis of variance (ANOVA) with Tukey’s post-hoc tests was used to identify which conditions were significantly different from each other at a significance level of *** *P*<0.001 and **** *P*<0.0001.

**Supplemental Figure 2. Single-cell RNA-Seq data demonstrating cell-type markers and cell type distribution.** (**A**) Examples of high expression of cell-type markers on the single-cell RNA-Seq tSNE plot and cell type distribution based on condition. *Ms4a1*, B cells; *Cd3g*, T cells; *Hbb*, erythroid cells; *Ahsp*, erythroid cells; *CD8a*, CD8 T cells; *Fcna*, macrophages; *Fcnb*; monocytes; *Nkg7*, NK cells. (**B**) Cell type distribution based on condition (KRV, uninfected, WT, *Ifnar1*^-/-^) in scRNA-Seq studies.

**Supplemental Figure 3. KRV does not infect WT rat pancreatic beta cells.** Two representative islets are shown from pancreatic sections from a KRV-infected LEW.1WR1 rat at 5 dpi. ISH probes for KRV (red), *Ins* (green), and *Gcg* (purple) were used. No KRV+ cells were seen in any islets.

**Additional supplementary data available:**

**Supplemental File 1**. (**A**) Excel worksheets for bulk seq DE analysis with GO analysis, scRNA-seq with select GO analysis, and KRV transcripts in scRNA-seq. Key: baseMean, mean expression level across all samples; log_2_FoldChange. log_2_ of the fold-change of the difference in expression level between the two conditions; lfcSE, the standard error of the log_2_FoldChange measurement; stat, the Wald statistic for the log_2_FoldChange, which takes the mean, logFC, and error into account; pvalue, the raw pvalue; padj, the Benjamini-Hochberg adjusted p-value which is corrected for multiple hypothesis testing.
